# Supplementary figures and images for: Comparative transcriptome analysis identified candidate genes associated with kernel row number in maize
Source: PeerJ. 2025 Mar 31;13:e19143. doi: 10.7717/peerj.19143 (PMC11967441; doi:10.7717/peerj.19143)

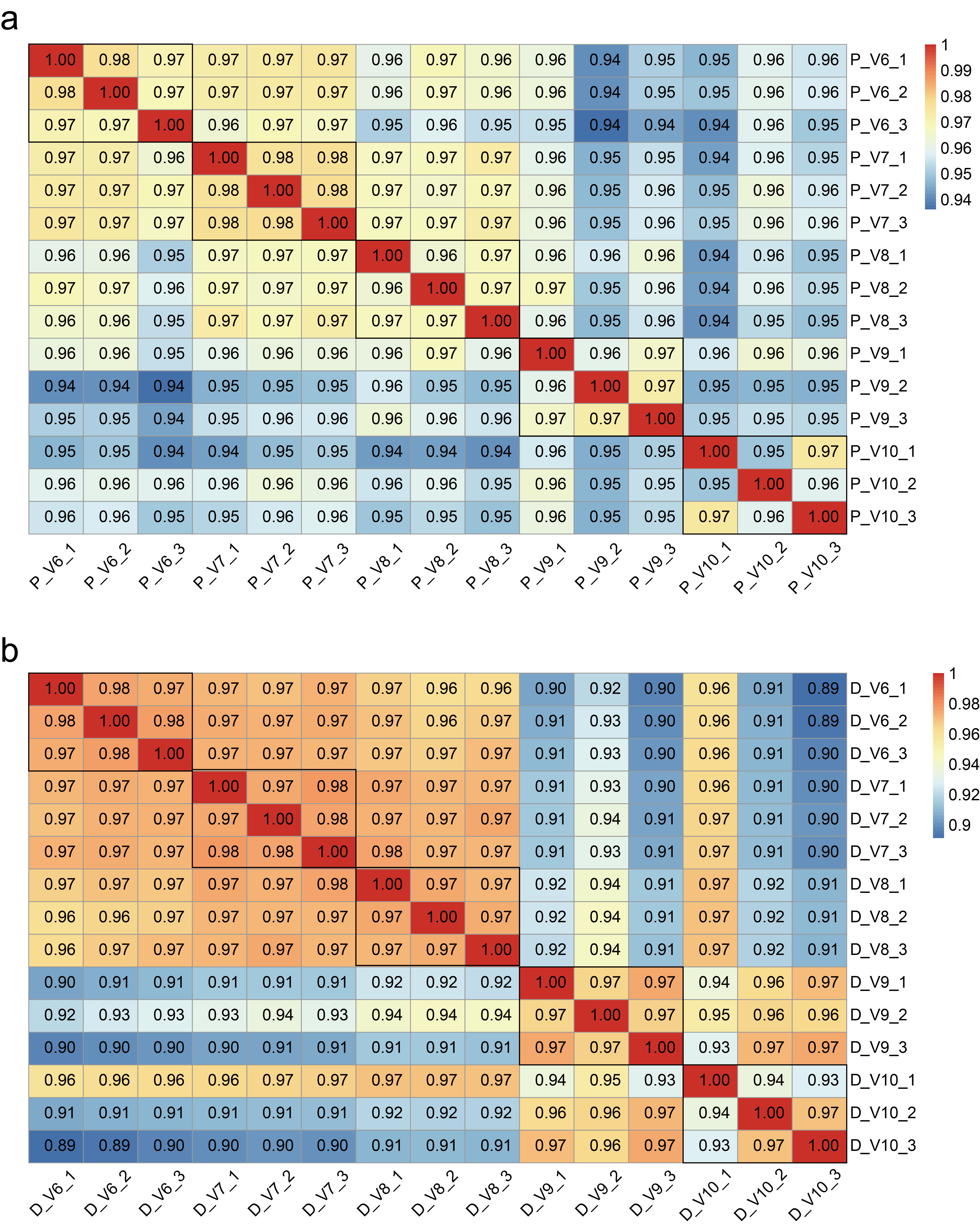

Supplement: Supplemental Information 1 — (A) SCC among the replicates of PHG35. (B) SCC among the replicates of Dan598. P, PHG35; D, Dan598; V, Vegetable stage; 1-3, three biological replicates. [file peerj-13-19143-s001.png]

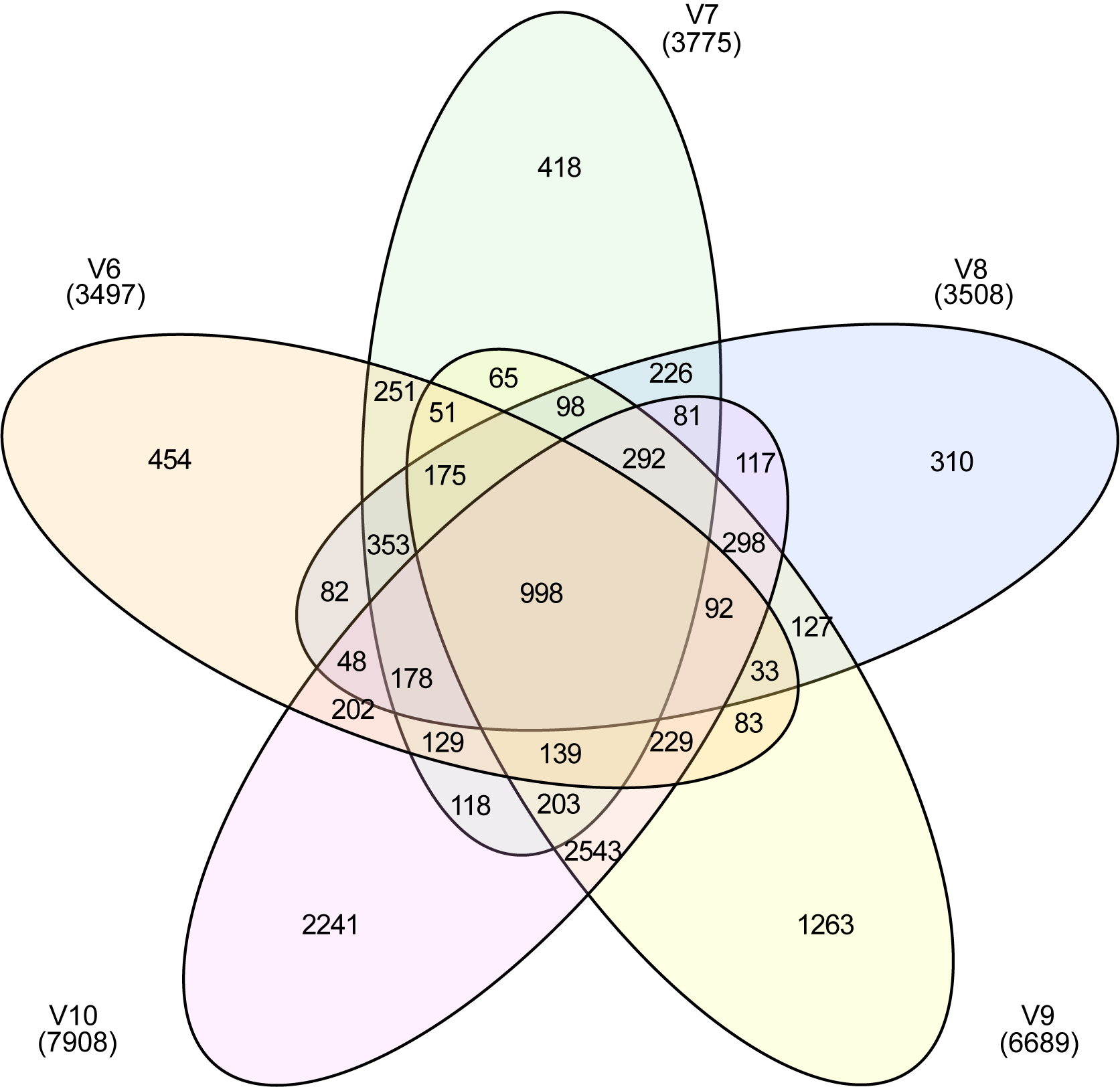

Supplement: Supplemental Information 2 — Screening of line-specific DEGs in inbred line Dan598 compared with PHG35 from V6 to V10. [file peerj-13-19143-s002.png]
