# Supplementary material for: Comparative transcriptome analysis identified candidate genes associated with kernel row number in maize
Source: PeerJ. 2025 Mar 31;13:e19143. doi: 10.7717/peerj.19143 (PMC11967441; doi:10.7717/peerj.19143)
Supplement: Supplemental Information 3 [file peerj-13-19143-s003.docx]

**Table S1** Primers used for qRT-PCR

| Gene | Forward Primer Sequences (5’-3’) | Reverse Primer Sequences (5’-3’) |
| --- | --- | --- |
| Zm00001d031451 | ACCCCTTGCGTCACGATAC | GTACGAGAAATCCAGCATGA |
| Zm00001d020430 | GCGACGGAGGCTTGTCTGTT | CTGCACGCTCCTATCCTCAACT |
| Zm00001d031068 | CTGGAGTACCTGCACATGATGG | AGGTCGAAGTCCGTGAGCAT |
| Zm00001d013895 | GATCACCAGAGAAAGGACGGG | AAGGGAGATTCTCGGAAGCAAG |
| Zm00001d034633 | GGCACATGGTTCAGTCTCACGC | AATCCATCTGCGCTTGATGGACC |
| Zm00001d017391 | AAATTCCCCTTTCGACTCTCCC | TAATAGGGCAAGGAACGGATCG |
| Zm00001d052442 | AGGGAAACGAAAGATAGCTCCG | GGTCAGCATGTTTGACAGATCG |
| Zm00001d020941 | ACGATGCTCTGCACCTGGT | GCTGGTGGTGGAATGGTTGAA |
