# Supplementary material for: Comparative transcriptome analysis identified candidate genes associated with kernel row number in maize
Source: PeerJ. 2025 Mar 31;13:e19143. doi: 10.7717/peerj.19143 (PMC11967441; doi:10.7717/peerj.19143)
Supplement: Supplemental Information 4 [file peerj-13-19143-s004.docx]

**Table S2 Overview of transcriptome sequencing data**

| Sample ID | Raw Reads (M) | Clean reads (M) | Total bases (G) | Q30 Bases(G)  ( Q30 Bases /Total bases ) | GC Content  (%) | Mapped Reads with Unique Loci (%) | Mapping Ratio(%) |
| --- | --- | --- | --- | --- | --- | --- | --- |
| D_V6_1 | 42.49 | 42.19 | 6.24 | 5.83 (93.4%) | 54 | 80.19 | 89.76 |
| D_V6_2 | 49.51 | 49.10 | 7.27 | 6.76 (93.0%) | 55 | 79.18 | 89.87 |
| D_V6_3 | 50.94 | 50.58 | 7.49 | 7.00 (93.4%) | 55 | 80.20 | 90.03 |
| D_V7_1 | 45.42 | 45.06 | 6.62 | 6.17 (93.2%) | 55 | 77.67 | 88.46 |
| D_V7_2 | 40.25 | 39.94 | 5.90 | 5.52 (93.4%) | 55 | 80.07 | 89.37 |
| D_V7_3 | 49.78 | 49.35 | 7.32 | 6.81 (93.0%) | 54 | 79.51 | 89.43 |
| D_V8_1 | 44.80 | 44.48 | 6.60 | 6.16 (93.4%) | 55 | 80.73 | 90.02 |
| D_V8_2 | 49.81 | 49.38 | 7.35 | 6.89 (93.2%) | 55 | 81.10 | 90.17 |
| D_V8_3 | 46.07 | 45.64 | 6.78 | 6.30 (92.9%) | 55 | 79.84 | 89.57 |
| D_V9_1 | 40.94 | 40.62 | 6.02 | 5.61 (93.2%) | 57 | 81.11 | 89.86 |
| D_V9_2 | 43.08 | 42.73 | 6.35 | 5.93 (93.4%) | 57 | 81.91 | 90.33 |
| D_V9_3 | 40.13 | 39.80 | 5.91 | 5.49 (93.0%) | 57 | 78.25 | 87.99 |
| D_V10_1 | 45.73 | 45.35 | 6.72 | 6.28 (93.4%) | 55 | 80.93 | 90.11 |
| D_V10_2 | 46.20 | 45.83 | 6.79 | 6.33 (93.2%) | 57 | 78.59 | 88.19 |
| D_V10_3 | 45.48 | 45.07 | 6.65 | 6.18 (92.9%) | 57 | 78.25 | 87.99 |
| P_V6_1 | 51.91 | 51.39 | 7.64 | 7.08 (92.8%) | 55 | 79.63 | 89.27 |
| P_V6_2 | 43.32 | 42.97 | 6.39 | 5.95 (93.1%) | 54 | 80.17 | 89.25 |
| P_V6_3 | 44.48 | 44.10 | 6.54 | 6.17 (94.2%) | 53 | 78.84 | 88.66 |
| P_V7_1 | 43.09 | 42.76 | 6.33 | 5.96 (94.2%) | 53 | 80.44 | 89.91 |
| P_V7_2 | 45.29 | 44.90 | 6.66 | 6.26 (94.0%) | 53 | 78.82 | 88.94 |
| P_V7_3 | 44.62 | 44.25 | 6.57 | 6.18 (94.1%) | 53 | 80.06 | 89.91 |
| P_V8_1 | 44.12 | 43.79 | 6.51 | 6.13 (94.2%) | 52 | 78.76 | 88.79 |
| P_V8_2 | 44.11 | 43.71 | 6.48 | 6.10 (94.1%) | 55 | 81.55 | 90.49 |
| P_V8_3 | 44.50 | 44.16 | 6.56 | 6.18 (94.2%) | 54 | 81.23 | 90.96 |
| P_V9_1 | 46.75 | 46.39 | 6.92 | 6.51 (94.2%) | 54 | 79.93 | 89.90 |
| P_V9_2 | 43.03 | 42.68 | 6.33 | 5.96 (94.1%) | 54 | 80.50 | 90.06 |
| P_V9_3 | 42.26 | 41.92 | 6.22 | 5.86 (94.2%) | 53 | 80.13 | 89.31 |
| P_V10_1 | 44.44 | 44.09 | 6.55 | 6.16 (94.2%) | 53 | 79.32 | 88.71 |
| P_V10_2 | 50.51 | 49.93 | 7.44 | 6.99 (94.0%) | 53 | 73.15 | 88.74 |
| P_V10_3 | 42.51 | 42.16 | 6.25 | 5.89 (94.2%) | 53 | 78.51 | 88.07 |

Notes: tissues are shown. D, Dan598; P, PHG35. V6-V10, young ears developmental stages; 1-3, three biological replicates. M represents a million. Q30 represents the percentage of nucleotides with a quality value ≥ 30.
